# Recent advances in hormone-based biopharmaceuticals for cancer therapy

**DOI:** 10.1186/s43046-026-00411-5

**Published:** 2026-09-21

**Authors:** Syahmi Nuqman Sarifulnazwan, Nur Alia Fatihah Mohd Tajuddin, Siti Nur Nadhirah Mohd Nasaruddin, Nur Malihah Imtiyaz Muhammad Mukmin, Engku Shifaa Nur Sahira Engku Abdul Malik, Muhammad Taher, Deny Susanti, Junaidi Khotib, Md Faiyazuddin, Riyanto Teguh Widodo, Muhammad Salahuddin Haris, Hendra Budiman

**Affiliations:** 1 International Islamic University Malaysia, Kuala Lumpur, Malaysia; 2 Airlangga University, Surabaya, Indonesia; 3 Saveetha Institute of Medical And Technical Sciences, Chennai, India; 4 University of Malaya, Kuala Lumpur, Malaysia; 5 University of Kuala Lumpur, Kuala Lumpur, Malaysia; 6 Universitas Muhammadiyah Klaten, Klaten, Indonesia

**Keywords:** Endocrine therapy, Oral SERDs., GnRH antagonists, Endocrine resistance, PROTAC technology

## Abstract

Hormone-dependent cancers, particularly estrogen receptor-positive breast cancer and androgen receptor-driven prostate cancer, contribute substantially to global cancer-related morbidity and mortality. Endocrine therapy has long been the mainstay of treatment for these tumors because of its specific mechanism and good safety profile. The long-term success of common hormone-based treatments is often undermined by resistance, both intrinsic and acquired, disease recurrence, and the activation of compensatory pathways that tumor cells have adapted to. To overcome these challenges, it has been necessary to develop high-quality hormone-based biopharmaceuticals that target the molecular level, enhance pharmacokinetics, and apply precision medicine strategies. The goal of this literature review is to provide an update on progress in hormone-based biopharmaceuticals for cancer management, with a particular focus on preventing endocrine resistance in hormone-dependent cancers. Among the treatments mentioned in the review, most are oral selective estrogen receptor degraders (SERDs) for ER-positive breast cancer, oral gonadotropin-releasing hormone antagonists for prostate cancer, genomically guided combination therapy that integrates inhibitors of the CDK4/6 and PI3K/AKT/mTOR signaling pathways, and next-generation androgen receptor (AR) degraders based on proteolysis-targeting chimera (PROTAC) technology. There is new evidence that these biopharmaceutical methods may be able to treat patients better over longer periods. For those who are less responsive, these approaches are even more effective when paired with proper biopharmaceutical methods, as in the case of hormone therapy. While targeted protein degradation and biomarker-driven combination therapies are repositioning drug development away from receptor blockade, the latter remains the mainstay of treatment. However, these developments do not eliminate concerns related to long-term safety, cost-effectiveness, appropriate patient selection, and resistance to novel agents. The path for hormone-based biopharmaceuticals in personalized cancer therapy requires continuous translational research and clinical validation to determine the best place.

## Introduction

Endocrine pathways are crucial as a source of effective cancer therapy approaches, as most cancers rely on the action of hormones, which account for a significant portion of cancer cases worldwide. This is because the proliferation of these malignancies is greatly influenced by hormonal signalling pathways. For instance, estrogen and androgen signalling play a vital role in breast and prostate cancer, respectively, as mentioned by Capper et al. [1], which favour hormonal pathways critical targets for therapeutic intervention. With high incidence and chronic disease courses, this will drive major cancer-related morbidity and mortality worldwide. As indicated by the World Health Organization (WHO), cancer ranks second among causes of death and contributes to an estimated 10 million deaths annually. As stated by Kristina et al. [2], breast cancer has the highest age-standardized incidence rate (ASIR) among the Association of Southeast Asian Nations (ASEAN). The most significant risk factors, including alcohol consumption, smoking, obesity, lack of physical exercise, and an unhealthy diet, contribute greatly to cancer incidence. The complexity of cancer, which involves how it arises and spreads, requires ongoing research and the development of advanced treatment strategies.

In this review, hormone therapy and endocrine therapy refer to treatments that suppress hormone production or inhibit hormone-receptor signalling. Hormone-based biopharmaceuticals refer to therapeutic strategies that modulate hormone-related pathways, including receptor degradation, androgen deprivation and targeted protein degradation. Targeted endocrine combinations refer to endocrine therapy combined with agents targeting resistance pathways such as CDK4/6 or PI3K/AKT/mTOR. Hormone therapy is sometimes referred to as endocrine therapy or hormonal therapy, according to the Journal Hormone therapy for cancer (2025). These approaches remain clinically important because they can provide durable disease control with generally favourable tolerability in hormone-responsive cancers. Wilcken et al. [3] stated that endocrine therapy is mainly given to women whose cancer is shown to be hormone dependent, meaning that the tumour cells regulate hormones like progesterone or estrogen receptors. In addition, biopharmaceuticals are large-molecule medications that comprise biosimilars, which are very similar to previously approved biologics, and biologics, which are protein-derived molecules cultivated in cells.

Although breast and prostate cancers remain the main clinical models for endocrine therapy, hormone-receptor signalling is also relevant in other malignancies. In endometrial cancer, ER and progesterone receptor (PR) signalling influence tumour biology and support the use of hormonal approaches such as progestins and aromatase inhibitors in selected patients. Hormone-related pathways have also been implicated in ovarian cancer, where progesterone receptor and other steroid receptor networks may contribute to tumour behaviour and therapeutic response. Therefore, hormone-based therapeutic strategies should be viewed as a broader concept involving ER, PR and AR signalling across different tumour types, although the strongest clinical evidence remains in ER-positive breast cancer and AR-driven prostate cancer.

According to Kumar et al. [4], Beatson (1848–1933) identified the initial use of hormone manipulation as a method of treating cancer, including breast cancer. This took place in 1895 with oophorectomy, or removal of the ovaries. The role of estrogen receptors in breast cancer was fully explored in the early 1950s with specific medications such as Tamoxifen. The goal of these medications is achieved through inhibiting the estrogen receptor. However, after some time, in 1990, trastuzumab was introduced as a result of further research in HER-2 positive breast cancer. Hormonal treatments, which remain fundamental in the management of breast cancers that test positive for hormone receptors, have come a long way and have been proven effective in some cases, better than Tamoxifen.

However, the long-term benefit of endocrine therapy is limited by disease recurrence, adverse effects and intrinsic or acquired resistance. Hence, biopharmaceutical hormones are created in parallel with advances in drug design and delivery, as well as an improved understanding of the complexity of hormone receptor signalling in order to find solutions to these challenges. This approach has significantly expanded the therapeutic options for patients with advanced or treatment-resistant cancer. It is also noted that most of these agents have portrayed promising clinical activity with improved progression-free survival and manageable safety profiles in clinical trials. Consequently, by bridging the gap between research discoveries and clinical application, the combination of genetic biomarkers and precision medicine strategies has enhanced patient selection and therapy tailoring [5].

Recent advances in endocrine and hormonal therapy are currently undergoing paradigm changes, shifting from conventional receptor antagonism to more complex biopharmaceutical interventions which utilize use of the internal functions of the cell. This could lessen the therapy’s effectiveness and safety. Currently, clinical focus is centred on various novel hormone-based biopharmaceuticals, including oral SERDs, oral GnRH antagonists, next-generation AR degraders, and PROTAC technologies, endocrine therapy combined with targeted agents, particularly CDK4/6 inhibitors and PI3K/AKT/mTOR pathway inhibitors, and strategies targeting resistance pathways, especially for the PI3K/AKT pathway. This review attempts to critically analyze recent advances in hormone-based biopharmaceuticals in cancer treatment, emphasizing their mechanisms of action, examples, clinical prospects, and unresolved challenges.

## Methodology

**Fig. 1:**
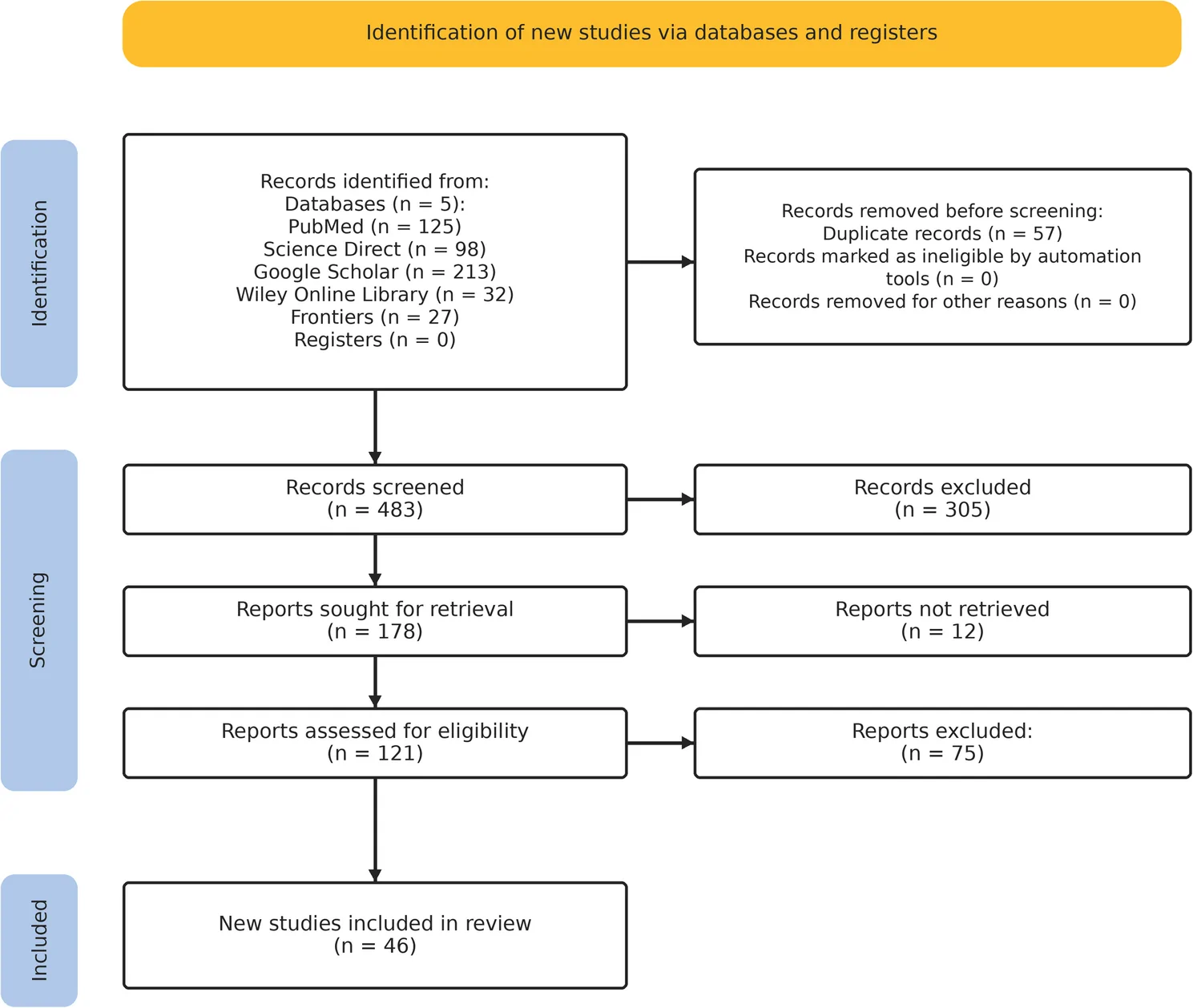
PRISMA 2020 flow diagram showing the identification, screening, eligibility assessment and final inclusion of studies in this review

To identify, evaluate, and synthesise the most recent data on the development of hormone-based biopharmaceuticals for cancer therapy, this literature review was conducted using a structured and systematic approach. Major electronic resources such as PubMed, ScienceDirect, Google Scholar, Frontier and Wiley Online Library were used to conduct a thorough search of peer-reviewed scientific literature. To include the most recent development in hormone-based biopharmaceuticals for cancer therapy, the search was focused on articles published between 2010 and 2025. Hormone-based therapy, endocrine therapy, biopharmaceuticals, breast cancer, prostate cancer, oral SERDs, GnRH antagonist, PI3K/AKT pathway, CDK4/6 inhibitors, AR degrader, and PROTAC technology were among the relevant keywords and Medical Subject Headings (MeSH) terms that were used in different combinations. Boolean operators such as “AND” and “OR” were used to improve the applicability of the result and enhance the search technique. Original research articles, systematic reviews, meta-analyses, and landmark clinical trials and studies focusing on hormone-dependent cancers, especially breast and prostate cancer, and lastly articles addressing molecular mechanisms, therapeutic efficacy, resistance pathways, or recent drug innovation are the predetermined inclusion criteria that were used to select the studies. The chosen articles were critically analysed to make sure the content is relevant in order to extract important details about the mechanism of action, clinical effectiveness, safety profile, resistance mechanism, and prospective future treatment. Data were then thematically organized according to major therapeutic advancements, including oral SERDs, GnRH antagonists, targeted combination therapies, AR degraders, and resistance-targeting pathways. This narrative synthesis method made it possible to combine clinical data with molecular insight to offer a thorough summary of both established and new hormone-based pharmacological approaches in cancer treatment (Fig. 1). 

The identification, screening, and inclusion of studies followed the PRISMA 2020 guidelines. A total of 495 records were identified through searches of five databases: PubMed (*n* = 125), ScienceDirect (*n* = 98), Google Scholar (*n* = 213), Wiley Online Library (*n* = 32), and Frontiers (*n* = 27), with no records identified from registers. After removing 57 duplicate records and no exclusions due to automation tools or other reasons, 483 records remained for screening. During the screening process, 305 records were excluded based on title and abstract review, leaving 178 reports sought for full-text retrieval. Of these, 12 reports could not be retrieved, resulting in 121 full-text articles assessed for eligibility. Following full-text evaluation, 75 articles were excluded for not meeting the inclusion criteria, and ultimately, 46 studies were included in the final review.

## Oral SERDs in ER-positive breast cancer

### Overview of estrogen receptor–positive breast cancer and endocrine therapy

ER-positive breast cancer accounts for most breast cancer cases, and ER signalling remains a major therapeutic target. Although endocrine therapy with aromatase inhibitors, selective estrogen receptor modulators and fulvestrant remains central to treatment, resistance and relapse have driven the development of newer oral SERDs.

Moreover, ER signalling serves as the main source of tumour development and long-term survival. Consistent with this, Kim and Lukong (2025) [6] reported that about 70% of breast cancers are ER-positive and that endocrine therapy remains the preferred first-line treatment.

### Limitations of conventional endocrine therapies and development of resistance

Despite initial benefit from conventional endocrine therapy, resistance may develop through ESR1 mutations, bypass signalling and adaptive cellular changes. Endocrine resistance can be broadly divided into early and late resistance. Early resistance is usually associated with intrinsic pathway activation, low ER dependence, growth-factor receptor crosstalk and ligand-independent ER signalling. In contrast, late resistance often develops after an initial response and may involve acquired ESR1 mutations, clonal selection, PI3K/AKT/mTOR activation, cell-cycle escape, epigenetic remodelling and tumour plasticity. This distinction is clinically important because early resistance may require upfront combination therapy, whereas late resistance may support biomarker-guided treatment adaptation.

These mechanisms reduce the long-term effectiveness of conventional endocrine therapies, including aromatase inhibitors, tamoxifen and fulvestrant. Resistance may arise through ESR1 mutations, activation of bypass pathways, ligand-independent ER signalling, and adaptive cellular changes. Tamoxifen resistance may additionally involve tissue-specific ER activity, activation of alternative transcriptional programmes such as AP-1, and growth-factor-mediated ligand-independent ER signalling, as discussed by Fanning and Greene [8]. Together, these mechanisms provide the rationale for oral SERDs as alternative agents with improved pharmacokinetics, greater patient convenience and potential activity in resistant ER-positive breast cancer [6, 7]. By binding to ER, promoting receptor degradation and suppressing estrogen-driven transcriptional activity, SERDs offer a strategy for overcoming endocrine resistance.

### Estrogen receptor biology and signalling pathways

Estrogen receptors (ERs) are steroid hormone receptors that function as ligand-activated transcription factors. The two main ER isoforms, ERα and ERβ, bind estrogen but have different roles in breast cancer biology. ERα is the major driver of ER-positive breast cancer, whereas ERβ is often reduced during carcinogenesis. Upon estrogen binding, ERs regulate transcriptional programmes involved in tumour cell survival and proliferation. These receptor-dependent pathways provide the biological basis for endocrine therapy and newer ER-degrading strategies.

According to Ascione et al. [9], the ESR1 gene on chromosome 6 encodes ERα and the ESR2 gene on chromosome 14 encodes ERβ, with both receptors present in normal mammary tissue. The authors also mentioned that ERs undergo structural changes that promote dimerization and translocation into the nucleus when estrogen binds to its receptors in the cytoplasm. Then, ER dimers bind to Estrogen response elements (EREs), which are the specific sequences of DNA that control transcription of estrogen-responsive genes that are involved in cell survival and proliferation.

Beyond canonical genomic signalling, steroid hormone receptors also function as dynamic signalling hubs. ER and AR can activate extranuclear pathways, including Src, PI3K/AKT, MAPK, PKC and FAK, which regulate chromatin remodelling, ROS-associated stress responses, cytoskeletal dynamics and adaptive survival [10–12]. These non-genomic mechanisms help tumour cells adjust their stress threshold under endocrine pressure and contribute to therapeutic resistance [13]. Therefore, endocrine resistance should be viewed not only because of receptor mutation or pathway activation, but also as an adaptive process involving tumour plasticity, stress-response signalling and compensatory survival networks. This broader perspective is also relevant beyond classical hormone-dependent cancers, as sex-related differences in immune responses and tumour biology may influence cancer development and treatment outcomes in malignancies such as melanoma and gastrointestinal cancers [14, 15].

### Classes of endocrine therapies targeting estrogen signalling

Current endocrine therapies for ER-positive breast cancer include aromatase inhibitors, SERMs and SERDs. Unlike SERMs, which may show tissue-dependent agonist or antagonist activity, SERDs promote ER degradation and more complete suppression of ER-driven signalling. This mechanism has supported the development of oral SERDs with improved convenience and potential activity in resistant disease. Neupane et al. [7] mentioned that SERMs like Tamoxifen work as antagonists. It also mentioned that they compete with estrogen for the ER binding site and inhibit the expression of genes involved in cell growth and proliferation. This distinction reflects the ability of SERDs to reduce receptor abundance through degradation rather than relying solely on receptor antagonism.

### Drug development and evolution of SERDs

For early breast cancer (EBC), SERMs and AIs are the current standard adjuvant endocrine therapy, in the presence or absence of ovarian function suppression, which depends on menopausal state and recurrence risk. AIs are also recommended treatment for postmenopausal patients. In addition, high-risk patients might benefit from advanced treatment such as neoadjuvant chemotherapy, including CDK4/6 inhibitors combined with endocrine therapy or adjuvant Olaparib, which is used in germline BRCA mutation carriers. Neoadjuvant endocrine therapy is typically reserved for patients who are ineligible for chemotherapy or have low-risk malignancies. There was no SERD approved for EBC at that time [16].

Fulvestrant, the first SERD authorized to treat metastatic hormone receptor-positive breast cancer, requires intramuscular administration and has limited bioavailability, motivating the development of second-generation oral SERDs with improved bioavailability and patient convenience. It successfully shows potential effectiveness, especially in cancers with ESR1 mutations, which is a common mechanism of endocrine resistance. The FDA has approved Elacestrant for metastatic breast cancer with ESR1 mutations that have worsened on earlier endocrine therapy.

According to Ascione et al. [9], the clinical studies have focused on two major SERD types. First, SERDs with acrylic acid side chains, GDC-0810 (Brilanestrant), AZD9496, and LSZ102, have demonstrated minimal efficacy in complete antagonistic activity and have an undesirable toxicity profile. Therefore, the research ended at an early stage. On the other hand, authors also mentioned that SERDs with amino side chains showed better clinical activity, reducing tumour cell proliferation by enhanced estrogen receptor inhibition with lesser toxicities. This class includes Amcenestrant, Camizestrant, Elacestrant, Giredestrant, and Imlunestrant. The primary toxicity concerns associated with oral SERDs are fatigue, nausea, and gastrointestinal adverse effects.

### Amcenestrant: preclinical promise and clinical failure

Preclinical studies show that second-generation oral SERDs are effective in treating ERα-positive breast cancer, including tumours with ESR1 mutations, which are a common cause of endocrine resistance. Pathak & Oliveira [17] mentioned that Amcenestrant is a nonsteroidal oral SERD that has shown good preclinical and early clinical outcomes as well as effectiveness against ESR1 mutations. However, further randomized trials did not demonstrate further benefits over regular endocrine therapy. Based on the research from the authors, the phase II AMEERA-3 research found no progression-free survival (PFS) advantage compared to the choice of physicians related to endocrine therapy, while the phase III AMEERA-5 trial was stopped after a poor preliminary evaluation. As a result, Amcenestrant’s clinical development has been terminated, which explains the limitations of translating preclinical SERD potency into meaningful therapeutic advantages.

### Elacestrant: first FDA-approved oral SERD

According to Guglielmi et al. [18], Elascestrant selectively binds to ERα and causes receptor degradation, which inhibits estrogen signalling. Unlike other SERDs, Elacestrant has both SERD and SERM characteristics with dose-dependent agonist or antagonist actions. In addition, at larger doses, Elacestrant acts as a pure antagonist and ER degradation agent. Pathak & Oliveira [17] also mentioned that Elacestrant is the first oral SERD approved by the Food and Drug Administration (FDA) and European Medicines Agency (EMA) to treat ER-positive and HER2-negative metastatic breast cancer with ESR1 mutations. It was approved based on the phase III EMERALD trial, which found a significant improvement in progression-free survival (PFS) compared to standard endocrine therapy, especially in patients with ESR1 mutations and a history of CDK4/6 inhibitor exposure. The authors also mentioned that Elacestrant had a controllable safety profile with mainly grade 1–2 gastrointestinal adverse reactions and relatively low withdrawal rates.

Moreover, Elacestrant has shown antiproliferative effects in neoadjuvant studies and is presently being assessed in several adjuvant and ctDNA-guided trials distinct from the metastatic setting to show its possible effectiveness at the beginning of the disease cycle. This also aligned with Pancholi et al. [19], which has mentioned that in vivo study, Elacestrant exhibits strong anticancer activity in a variety of patient-derived xenograft (PDX) models with common ESR1 mutations such as Y537S, Y537N, and D538G which include fulvestrant-resistant tumours where fulvestrant is ineffective or shows minimal efficacy. When Elacestrant is combined with CDK4/6, PI3K, or mTOR inhibitors, combination trials show additive or synergistic antitumor outcomes, and Elacestrant maintains action in palbociclib-resistant models, as explained by Pancholi et al. [19].

### Giredestrant: potent preclinical activity with variable clinical results

Moreover, according to Guglielmi et al. [18] and Pathak & Oliveira [17], both explained that Giredestrant is a very potent oral SERD that causes robust ER antagonism and entire receptor degradation in MCF-7 cells, which results in 100% ER loss at sub-nanomolar dosages and tumour suppression in preclinical studies. They also mentioned that early-phase clinical studies indicated potential activity, particularly when combined with CDK4/6 or PI3K inhibitors. However, the phase II acelERA trial failed to show further advantages over regular endocrine therapy in an unselected group. Exploratory analysis and neoadjuvant studies suggest that Giredestrant is more effective in ESR1-mutant illness.

Recent phase III lidERA data have strengthened the clinical relevance of giredestrant as a novel oral SERD in ER-positive/HER2-negative early breast cancer. In this global randomized trial, adjuvant giredestrant significantly improved invasive disease-free survival compared with standard endocrine therapy in patients with stage I–III disease. At the interim analysis, giredestrant reduced the risk of invasive disease recurrence or death, with a favourable safety profile and fewer treatment discontinuations than standard endocrine therapy. These findings suggest that giredestrant may become an important adjuvant endocrine option, although longer follow-up is required to confirm overall survival benefit and define the patients most likely to benefit.

### Imlunestrant: combination potential and ongoing development

Next, Imlunestrant is an oral SERD that has robust ER antagonist and degradative characteristics, as well as favourable pharmacokinetics. According to Guglielmi et al. [18], Imlunestrant is an orally bioavailable SERD which has nanomolar affinity for ERα and mutant variations like Y537N. In preclinical studies, it promotes long-term ER inhibition, reduces tumour development and works as a pure antagonist with no uterotrophic effects. The authors also discussed that Imlunestrant is particularly useful in combination therapy since it has additive or synergistic antitumor effects when combined with target agents including CDK4/6, mTOR and PI3K inhibitors.

This aligned with Pathak and Oliveira [17], who explained that in the phase I EMBER research, Imlunestrant showed consistent clinical activity in strongly pretreated patients, including those with recent CDK4/6 inhibitor exposure and ESR1 mutations, and with a low frequency of grade ≥ 3 side effects. Combination cohorts with Abemaciclib had significantly greater response rates, showing a synergistic interaction. Pathak and Oliveira [17] also mentioned that preoperative research revealed significant decreases in Ki-67 and ER expression, which suggests successful biological activity, and Imlunestrant is now being investigated in phase II and III trials in both metastatic and adjuvant clinical settings.

Recent EMBER-3 phase III data provide stronger clinical evidence for imlunestrant in ER-positive/HER2-negative advanced breast cancer. In patients with ESR1-mutated disease, imlunestrant improved progression-free survival compared with standard endocrine therapy, although this benefit was not significant in the overall study population. The combination of imlunestrant with abemaciclib further improved progression-free survival compared with imlunestrant alone, supporting its potential role as part of targeted endocrine combination therapy [19,21].

### Camizestrant: advanced clinical development

Last but not least, Camizestrant is an ERα antagonist that promotes ER degradation and inhibits ER-mediated signalling. It has a sub-nanomolar ER binding affinity and a degradation activity similar to Fulvestrant in ER-positive breast cancer cell lines. Lawson et al. [22] mentioned that Camizestrant showed potent and selective ER degradation, altered ER-regulated gene expression and produced total ER antagonism and noticeable antiproliferation action in ESR1 wild-type (ESR1wt) and mutant (ESR1m) breast cancer cell lines and patient-derived xenografts (PDX). In Fulvestrant-resistant wild-type (normal) Estrogen Receptor 1 gene (ESR1wt) and wild-type (mutant) Estrogen Receptor 1 gene (ESR1m) PDX models, Camizestrant also demonstrated potent antitumor efficacy. Furthermore, in CDK4/6-naive and -resistant models, Camizestrant was evaluated in combination with CDK4/6i (Palbociclib or Abemaciclib), PI3Kαi (Alpelisib), mTORi (Everolimus) or AKTi (Capivasertib). The results explained by the authors which Camizestrant was active when combined with CDK4/6i or PI3K/AKT/mTORi and that the triple combination further enhanced its antitumor action.

Moreover, according to Pathak and Oliveira [17], in the phase II SERENA-2 trial, Camizestrant at 75 mg and 150 mg doses greatly enhanced PFS compared to Fulvestrant, particularly in patients with ESR1 mutations as well as patients who had been treated with CDK4/6 inhibitors. Authors also mentioned that despite certain risks, including asymptomatic sinus bradycardia and low-grade visual abnormalities, Camizestrant is usually well tolerated. Camizestrant is presently being studied in broad phase III trials in both metastatic and adjuvant scenarios according to the findings. This is supported by Turner et al. [23], which in the study, SERENA-6 is a phase III clinical research that will determine if changing from an Aromatase Inhibitor to Camizestrant while maintaining the same CDK4/6 inhibitor able to improve survival in patients with HR+/HER2- advanced breast cancer who develop ESR1 mutations. The goal of the research is to minimize the demand for chemotherapy and optimize estrogen-driven tumour growth.

Taken together, the SERENA studies position camizestrant as a biomarker-guided endocrine strategy, particularly in ESR1-mutated disease.

Oral SERDs have potential for treating ER-positive breast cancer, but they should not be viewed as a uniform drug class. Their clinical outcomes may differ due to variations in receptor binding, degradation potency, pharmacokinetics, toxicity, ESR1-mutant activity and compatibility with targeted combinations. The clinical failure of amcenestrant despite promising preclinical activity highlights the translational gap between experimental efficacy and patient benefit. Although oral SERDs offer improved convenience compared with injectable fulvestrant, their tolerability profiles require careful consideration. Commonly reported adverse effects include nausea, vomiting, fatigue, hot flushes, gastrointestinal discomfort and elevated liver enzymes, while selected agents may also show cardiovascular effects such as bradycardia. Therefore, treatment selection should consider ESR1 mutation status, prior therapy, pathway activation, adverse-effect profile, comorbidities, drug tolerability, patient preference and the availability of targeted combination strategies [24, 25].

### Oral GnRH antagonists

Prostate cancer is predicted to cause 499,000 deaths and 1.7 million cases by the year 2030, as cited by Sekhoacha et al. [26]. The aetiology of prostate carcinoma involves various aspects in terms of genetic, epigenetic, inflammatory mediators, and biological factors which interact in a complex manner. Androgen-related factors, such as signalling and metabolism, show intimate correlation with carcinogenesis and tumour growth. In addition, various genetic and epigenetic alterations, such as methylation and mutations of genes, provoke it. In addition to other factors like comorbidity, baseline urinary function, and age, other specific prognostic factors namely beginning level of PSA, clinical TNM stages, together with Gleason’s grade, have been taken into account in the scope of achieving a treatment for prostatic carcinoma.

According to Huggins and Hodges’ 1941 discovery, the overproduction of testosterone, which is an androgen hormone, is typically the cause of prostate cancer [27]. As a result, the researchers created a treatment that uses endocrine manipulation as a fundamental therapeutic approach. Androgen deprivation therapy (ADT) is one method of reducing or inhibiting this hormone production, which acts by stopping the proliferation of cancer cells. Oral Gonadotropin Releasing Hormone (GnRH) antagonists were created as a result to lower testosterone levels in the blood. Oral GnRH antagonists are unique because they eliminate the initial spike in testosterone that agonists induce. Injectable peptide GnRH agonists have historically been developed in which they work by overstimulating the pituitary gland, causing a brief spike in testosterone and Luteinizing Hormone (LH), known as a “testosterone flare,” before suppression begins, which exacerbates cancer patients’ symptoms [28].

The significant increase in testosterone production happened when leuprolide and other GnRH agonists activate GnRH receptors in the hypothalamus, leading to stimulation of the anterior pituitary gland to secrete LH and Follicle Stimulating Hormone (FSH). Initially, there is a significant increase in testosterone and dihydrotestosterone production, resulting in a “flare effect” and temporary regression of prostate cancer. Constant activation of GnRH receptors results in desensitization and downregulation, which lowers testosterone, FSH, and LH. This shows that antagonists do not cause the hormonal surge, leading to rapid suppression [29]. Moreover, Stangelberger et al. [30] also mentioned that short-term non-steroidal anti-androgen medication, such as flutamide and bicalutamide, may halt the flare phenomena. According to [31, 32, 33], some GnRH antagonists are frequently administered orally, show a rapid onset of action, and do not induce the flare-up effect, thereby overcoming key limitations of GnRH agonists and expanding current treatment options.

Shore [34] provides relugolix as a clinical example of this GnRH antagonist approach. Relugolix, an oral GnRH antagonist, can reduce testosterone levels and stop the pituitary gland from generating FSH and luteinizing hormone, as mentioned by [34]. Due to its testosterone-flaring effect, Relugolix offers cancer patients improved side effects, making it a better treatment option for them.

Since 1972, many GnRH antagonists and related animal studies have been conducted. It is clear that the first development of hydrophilic GnRH antagonists resulted in the release of histamine, which caused severe allergic reactions and transient oedema. D-ureidoalkyl amino acids were used to create new counterparts that eliminated this adverse effect of oedema. Cetrorelix was therefore the first drug in this class to be studied in prostate cancer patients. Despite showing significant clinical performance, cetrorelix was unable to access the market due to similar side effects and challenges in producing long-acting formulations. In 2003, the US Food and Drug Administration (FDA) approved the development of abarelix, the first GnRH antagonist used to treat prostate cancer. Unfortunately, it produced similar adverse reactions and was subsequently discontinued. Degarelix, a next-generation GnRH antagonist that showed reduced histamine release characteristics compared to previous generations, was subsequently developed and approved by the FDA. Relugolix (TAK-385), a small molecule non-peptide drug that exhibited acceptable safety and efficacy, is one of the established oral agents. However, another advance in prostate cancer ADT may be expected in the coming years. Liu et al. [29]) explain the history of GnRH receptor antagonists.

Additionally, Tatenuma and Miyamoto [35] found there were fewer instances of cardiovascular adverse events for the oral GnRH antagonist degarelix than the GnRH agonist, which could potentially have some adverse reactions, including the regression of the prostate cancer, when reported through the temporal association of the regression of the prostate cancer at the site of the injections. Hot flashes, headaches, and nausea consisted of the adverse reactions associated with Relugolix. Furthermore, according to [36], the QT interval was prolonged to more than 450 ms but not exceeding 480 ms. Several other unexpected and significant adverse event signals not recorded in the regulatory investigation were found in the current study, including erectile dysfunction, decreased libido, male genital atrophy, testicular atrophy, and gynecomastia. These findings from clinical trials and empirical research all indicate that testosterone suppression is a major factor contributing to the emergence of new adverse events. On the other hand, it is also promising that relugolix’s fast testosterone suppression and recovery rate may help in reducing the duration of drug exposure and decrease these adverse events. Clinical blood testosterone monitoring and efficient control can be used in Relugolix therapy as an intervention to reduce the risk of adverse events.

The financial benefit of Relugolix as a therapy for prostate cancer has also been examined. Based on a recent US study, the estimated monthly cost of ADT with Relugolix was $2866.94, more than five times higher than that of leuprolide ($602.99) [37]. Based on a later study, relugolix’s incremental cost-effectiveness and quality-adjusted life-year (QALY) ratio is 0.46, or $49,571.10/QALY. Relugolix and leuprolide exhibited relative rates of progression-free survival/overall survival at five years of 72.7%/86.0% and 61.0%/85.9%, respectively, when administered as first-line therapy. Although the influence of adverse events was not considered in these studies, this suggests that ADT with Relugolix may not be a more cost-effective alternative for the treatment of advanced prostate cancer compared to leuprolide. However, considering its decreased risk of cardiovascular adverse effects and the safety benefits of GnRH antagonists, Relugolix might still be recommended in some patients.

## Next-generation AR degraders and PROTAC technologies

Prostate cancer development and its progression are strongly associated with AR signalling. Androgen receptors are activated by testosterone and Dihydrotestosterone (DHT). Once activated, they regulate genes that control growth for normal prostate growth and function. In prostate cancer, there is an increase in the levels of both Androgen receptor protein and mRNA. Androgen receptor gene amplification occurs in 20–33% of cases of castration resistance. Higher Androgen receptors will make the cells hypersensitive even to low levels of androgen. Mutation of Androgen receptor occur mostly in ligand-binding domain, which make AR promiscuous, meaning it can be activated by weak androgens, other steroid hormone, anti-androgen like flutamide, which can paradoxically act as agonist that cause the development of resistant. Other than that, intratumoral androgen production by tumour cells. Tumour cells start making their own androgen, intrauterine androgen synthesis, even after medical castration, cancer will internally produce enough ligand to activate AR. There are also AR coactivator overexpression, these amplify Androgen receptor -transcriptional activity which can lead to progression of castration resistance. Some AR splice variants like AR-V7 lack the ligand-binding domain, so they are constitutively active even when there is no ligand binding, this is the major cause of castration-resistance prostate cancer [38].

Although prostate cancer is primarily driven by androgen receptor (AR) signalling, estrogen receptor biology is also relevant. The prostate expresses both ERα and ERβ, and these receptors can exert different biological effects. ERα has generally been associated with proliferative and pro-inflammatory signalling, whereas ERβ is often considered to have tumour-suppressive functions through regulation of differentiation, apoptosis, anti-proliferative signalling and interaction with AR-associated pathways. However, the role of ERβ is complex because ERβ isoforms and context-dependent signalling may contribute differently to tumour progression and therapeutic response. This distinction is clinically important when considering broad or non-selective anti-estrogen receptor strategies. Approaches that inhibit or degrade ERα without sufficient selectivity could theoretically affect ERβ signalling, which may be undesirable if ERβ retains protective or tumour-suppressive activity in a given prostate cancer context. Therefore, future endocrine-based approaches in prostate cancer should distinguish ERα from ERβ biology and consider receptor subtype selectivity when evaluating therapeutic benefit and risk.

AR biology should also be considered beyond prostate cancer. AR expression has been described in subsets of breast cancer, particularly molecular apocrine and AR-positive triple-negative breast cancer, where AR signalling may influence tumour growth, metastatic behaviour and response to targeted therapy. In endometrial cancer, emerging evidence also suggests interactions among ER, PR and AR signalling, indicating that AR may contribute to tumour progression and endocrine responsiveness in selected contexts.

According to Chen et al. [39], there are three major groups of drugs designed to destroy the AR. These new strategies are developed to overcome the castration-resistant prostate cancer (CRPC), and advanced prostate cancer where the cancer keeps growing even when the amount of testosterone in the body is reduced to very low levels. The first category consists of hydrophobic tagging degraders (HyT). These molecules function by attaching a bulky, hydrophobic group such as adamantyl – to an AR ligand. This modification causes the AR to mimic a misfolded protein, triggering the cell’s internal quality control systems to eliminate it. The second type is Monomeric AR degraders, which are small, single molecules that do not need two ligands, unlike PROTAC. They include UT-series, selective AR degraders such as UT-34 (ONCT-534) that can degrade full length AR and AR-V7, chaperone disruptors that interfere with proteins that protect AR like ASC-J9, Niclosamide, and Geldanmycin. Lastly, proteolysis-targeting chimeras (PROTACs) form the third group and are discussed in more detail below.

**Fig. 2:**
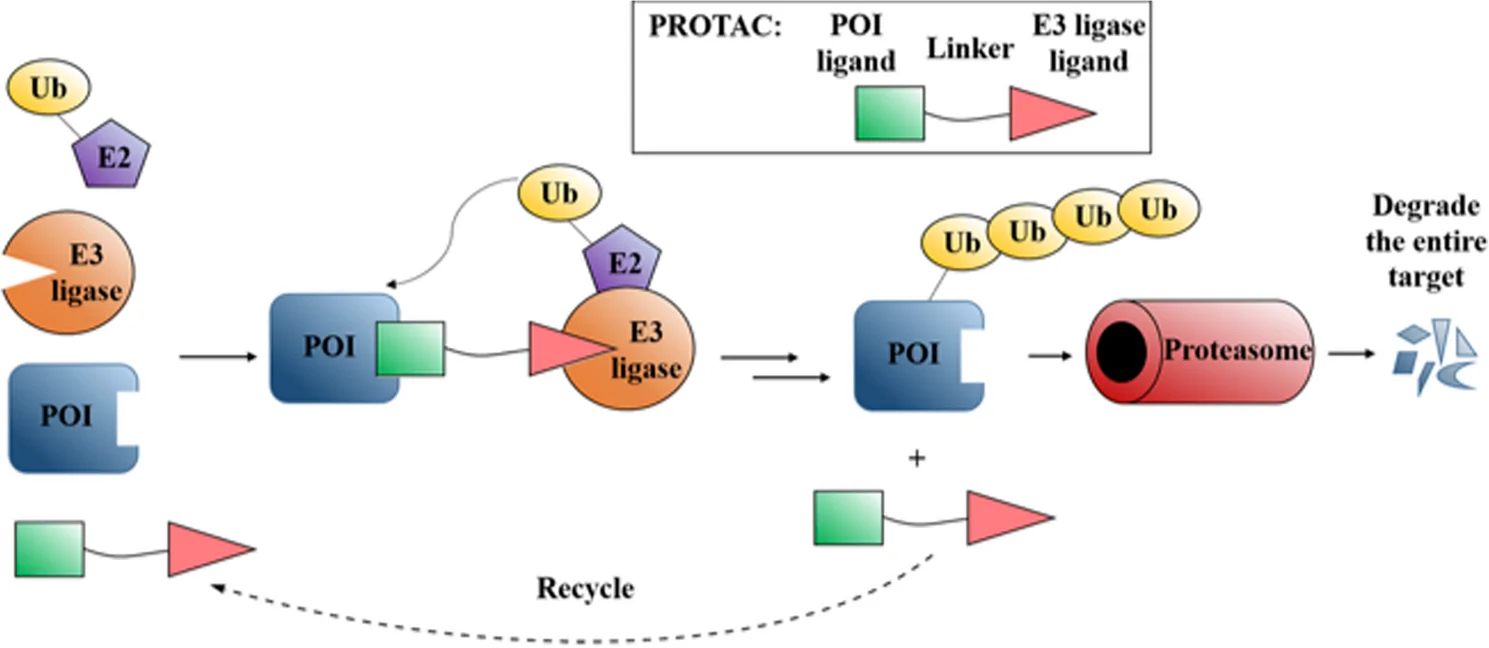
Mechanism of PROTAC-mediated protein degradation adapted from [41]

Kregel et al. [40] reported that newly designed AR degraders are capable of overcoming the resistance mechanisms that arise during prostate cancer therapy. The study demonstrates that AR protein degradation using PROTAC technology can overcome resistance to other AR-targeted therapies by using the new AR degrader compound, ARD-61. ARD-61 is an AR degrader 61, a proteolysis chimera (PROTAC) that degrades the full length AR and mutant forms. The mechanism of action is by binding of ARD-61 to AR, then ARD-61 simultaneously recruits the VHL E3 ubiquitin ligase, VHL attaches ubiquitin tags to AR, these tags send AR to proteasomes, which break down and eliminate the AR protein. Sun et al. [41] described that PROTACs represent a transformative approach to modulating proteins of interest (POIs) through targeted degradation. These heterobifunctional molecules utilize an optimized linker to bridge a POI ligand with an E3 ubiquitin ligase recruiting ligand. By facilitating the formation of a ternary complex, PROTACs bring the POI into close proximity with the cellular ubiquitination machinery. This triggers POI ubiquitination and subsequent degradation by the 26 S proteasome, leveraging the eukaryotic ubiquitin-proteasome system (UPS) (Fig. 2).

ARD-61 is much more potent than traditional AR drugs such as enzalutamide, which only block AR activity while ARD-61 removes the AR protein entirely. The advantages of this ARD-61 are greater inhibition of cancer cell growth. When prostate cancer cells are treated with ARD-61, their growth rate is much slower and has 100 times lower IC50 values, meaning that it is more potent as less drug is needed to achieve the desired response. Other than that, ARD-61 induces the cell apoptosis which can be seen by more PARP cleavage. Lastly, a larger reduction in AR-dependent genes such as PSA(KLK3), TMPRSS2, and NKX3.1, these genes are controlled by the AR, thus their reduction indicates ARD-61 degradation.

Zou et al. [42] describe PTOTAC technology as an innovative drug development that targets the ubiquitin proteasome system to eliminate proteins that cause the disease. PROTAC molecules have 3 essential components in their structure, the first part is the target protein ligand that binds specifically to the protein of interest, the E3 ubiquitin ligase ligand that binds to a specific E3 ubiquitin ligase, and lastly the linker moiety that connects the two ligands and determines spatial orientation. PROTAC enables event-driven pharmacology rather than occupancy-driven inhibition. Meaning that binding triggers permanent and complete degradation of the target protein. A single PROTAC molecule can induce degradation of multiple protein copies, enhancing its pharmacodynamic efficiency. PROTAC is useful in overexpressed protein, functional mutant protein and non-enzymatic scaffolding proteins.

According to Giovannelli et al. [43], ARs are expressed in 50–70% of all breast cancer, meaning breast tumours respond directly to androgen. So, androgen plays a crucial role in breast cancer cell growth, spread and making them an important target for treatment. Androgen antagonize estrogen driven proliferation in estrogen receptor (ER) positive tumours but act as direct oncogenic drivers through AR mediated growth signalling in ER negative and triple negative breast cancer, thereby the biological effect of androgen in breast cancer is determined by estrogen receptor status. Therefore, androgen can promote cancer growth via two pathways, which is the direct effect via AR that acts like a transcription factor that activates the gene that promotes cell proliferation, survival and invasion. The other pathway is an indirect effect; androgen is converted to estrogen by aromatase. Estrogen binds estrogen receptors that activate estrogen-driven growth pathways, which stimulate tumour proliferation.

Wang et al. [44] discuss PROTAC technology as an innovative approach for targeting various proteins that are responsible for breast cancer development. PROTACs are bifunctional molecules that selectively degrade proteins by attaching a target protein to an E3 ubiquitin ligase. Breast cancer progression is commonly caused by molecular targets such as ER, HER2, CDK4/6, PARP, BET proteins, EZH2, ARs, and PROTAC have been designed for targeting each of these. ER-targeting PROTAC such as ERD-308, ZD12, and TVHL-1 show strong anti-tumour activity which is effective against tamoxifen-resistant or ER-mutant cells. Other than that, CDK4/6 PROTACs that are derived from palbociclib show strong anti-proliferative activity with minimal toxicity. In addition, PARP targeting PROTACs such as NN3 and C8 are being developed to overcome the current PARP inhibitor that faces many limitations. Lastly, HER2 PROTACs such as CH7C4 work by degrading HER2 and suppressing the downstream signalling, which is effective for patients who are resistant to trastuzumab. PROTAC therapy for breast cancer is rapidly advancing but faces a few challenges, such as large molecular weight, poor oral absorption, and side effects. Therefore, new drug delivery innovations, including nanoparticles, aptamer-guided targeting, and light-activated PROTAC, are currently being developed to increase specificity and reduce the undesirable side effects.

Among ER-targeting PROTAC degraders, vepdegestrant should also be considered among the most clinically advanced ER-targeting PROTAC degraders. In the phase III VERITAC-2 trial, vepdegestrant was compared with fulvestrant in patients with ER-positive/HER2-negative advanced breast cancer previously treated with endocrine therapy and a CDK4/6 inhibitor. The trial showed improved efficacy in the ESR1-mutant subgroup, although the benefit was less definitive in the overall study population. This finding highlight both the promise and limitation of PROTAC-based ER degradation, particularly the need for biomarker-selected patient populations [45].

Breast cancer is one of the major causes of mortality among women, estrogen receptor (ER) and progesterone receptor (PR) play a major role in breast cancer development, but recent evidence also shows that AR also plays an important role in breast cancer progression. Zhao et al. [46] mentioned that ARD-61, a highly potent PROTAC AR degrader that efficiently inhibits AR-positive breast cancer growth in vitro and can suppress tumour growth in vivo. This study focuses on investigating the ARD-61 that completely eliminates AR rather than merely suppressing its activity. It is proven that ARD-61 effectively induces and almost completely degrades AR in AR-positive breast cancer. ARD-61 is also a potent inhibitor of cell growth, cell cycle arrest at G2/M phase and apoptosis in cell lines with high AR expression, such as MDA-MB-453 and HCC1428. In addition, in mouse xenograft models, a single ARD-61 dose can degrade AR protein in tumours and reduce downstream oncogenic signals, including c-Myc and WNT7B. Therefore, treatment with ARD-61 shows promising tumour-suppressing activity, achieving partial regression without major toxicity. These findings support ARD-61 as a new therapeutic approach for AR-positive breast cancer.

## Genomically guided targeted combinations with endocrine therapy

The estrogen receptor (ER) signalling pathway is central to the development and progression of ER-positive breast cancer. Although endocrine therapies effectively inhibit ER signalling, their clinical benefit is often limited by intrinsic or acquired resistance. Several mechanisms contribute to endocrine resistance, including dysregulation of ER pathway components, altered cell-cycle control, survival signalling and activation of alternative escape pathways that sustain tumour growth. Enhanced expression or activation of growth factor receptor pathways, especially the EGFR/HER2 signalling axis, has also been strongly linked to endocrine therapy resistance in experimental and clinical settings [47]. Therefore, combination strategies aim to suppress both ER signalling and compensatory escape pathways. However, clinical studies also emphasize the need to identify patients whose tumours are most likely to benefit from such co-targeting approaches [47].

One of the most extensively studied genomically guided combination strategies in hormone-receptor-positive cancer is the integration of CDK4/6 inhibitors with endocrine therapy. The cycling D-CDK4/6 pathway plays a central role in regulating cell cycle progression. Its dysregulation is commonly observed in hormone-related tumours, particularly ER-Positive Breast Cancer. According to [48], clinically approved CDK4/6 inhibitors such as palbociclib, ribociclib, and abemaciclib have shown substantial efficacy when combined with standard endocrine agents like aromatase inhibitors or SERDs. This significantly improves progression-free survival compared to endocrine therapy alone. However, a significant proportion of patients exhibit intrinsic or acquired resistance to CDK4/6 inhibitors, highlighting the complexity of tumour adaptive mechanisms under treatment pressure. Research indicates that resistance to CDK4/6 inhibitors may involve both cell cycle-specific escape mechanisms and activation of alternative signalling pathways. This includes those downstream of receptor tyrosine kinase and the PI3K-AKT-mTOR axis, which can undermine single-agent efficacy. Hence, combining CDK4/6 inhibitors with targeted endocrine therapy is not only guided by the molecular role of cell cycle control in hormone-related cancers but also reflects an effort to overcome genomic and signalling adaptations that contribute to previous treatment failures.

Targeting the PI3K/AKT/mTOR axis with endocrine therapy is particularly relevant in tumours with pathway activation, such as PIK3CA-mutated breast cancer. Combined inhibition may reduce compensatory AKT reactivation and restore endocrine sensitivity. The PI3K/mTOR pathway controls key processes including cell growth, proliferation, and survival, and its constituent activation has been implicated in cancer progression and resistance to hormone-based treatments. According to Yang et al. [49], Preclinical evidence demonstrates that dual inhibition of PI3K and mTOR can produce greater antitumor activity than inhibiting either component alone, as combined blockade more effectively suppresses downstream effectors and prevents compensatory reactivation of AKT signalling, which commonly limits the efficacy of single agents. Furthermore, combining PI3K/mTOR pathway inhibitors with endocrine agents, for example, tamoxifen or Fulvestrant, has shown potential to enhance endocrine responsiveness and overcome resistance in estrogen receptor-positive models by reducing signalling feedback mechanisms that drive cell survival despite hormone deprivation. This combined targeting strategy aligns with the genomic rationale of precision oncology, leveraging molecular alterations in the PI3K/AKT/mTOR pathway to select patients who may benefit most from such tailored therapeutic regimens.

Targeted triplet therapy has emerged as an important strategy for endocrine-resistant, hormone receptor-positive/HER2-negative advanced breast cancer. The phase III INAVO120 trial evaluated inavolisib, a PI3Kα inhibitor, in combination with palbociclib and fulvestrant in patients with PIK3CA-mutated advanced breast cancer who relapsed during or shortly after adjuvant endocrine therapy. The inavolisib triplet significantly improved progression-free survival compared with placebo plus palbociclib and fulvestrant. Updated analysis also showed an overall survival benefit, supporting this regimen as a clinically relevant example of biomarker-selected triplet therapy. However, the benefit was accompanied by increased adverse effects, including hyperglycaemia, stomatitis or mucosal inflammation, gastrointestinal toxicities and ocular toxicities, highlighting the need for careful patient selection and toxicity monitoring [20].

After that, beyond cell-cycle and growth factor pathway alterations, genomic mutations in the estrogen receptor gene (ESR1) itself represent a critical mechanism driving resistance to endocrine therapy and guiding targeted combination strategies. ESR1 encodes estrogen receptor-α, the central transcriptional driver in approximately 70% of breast cancers, and somatic mutations in its ligand-binding domain, most commonly Y537S and D538G, are frequently acquired following prolonged aromatase inhibitor exposure. Based on research conducted by Badhe [50], these mutations confer ligand-independent receptor activation, enabling persistent estrogen signalling even in estrogen-depleted conditions, thereby rendering aromatase inhibitors ineffective. Multi-omics and network analyses further demonstrate that ESR1 functions as a highly connected signalling hub, exhibiting extensive crosstalk with the PI3K/AKT/mTOR and cell-cycle pathways, which amplifies tumour adaptability and therapeutic escape. This genomic insight has driven the development of next-generation endocrine agents, including oral SERDs and PROTAC-based degraders, which directly target mutant ESR1 proteins. Importantly, these agents are increasingly evaluated in combination with CDK4/6 or PI3K pathway inhibitors, reflecting a genomically guided strategy aimed at simultaneously suppressing estrogen receptor signalling and downstream adaptive pathways to overcome resistance and improve clinical outcomes.

Combating endocrine therapy resistance requires personalised treatment selection based on tumour biology, prior treatment exposure and molecular biomarkers. ESR1 and PIK3CA mutation testing can guide the use of oral SERDs, PI3K pathway inhibitors and targeted endocrine combinations, while circulating tumour DNA monitoring may support earlier treatment adaptation before clinical progression. This approach helps match therapy to the dominant resistance mechanism and may reduce unnecessary toxicity.

Beyond treatment selection, genomic profiling can support therapeutic discovery by revealing recurrent genomic changes, pathway dependencies and mechanisms of tumour evolution under endocrine pressure. It should therefore be viewed not only as a diagnostic tool, but also as a platform for developing and refining biomarker-driven endocrine therapies in ER-positive breast cancer.

Overall, genomically guided combinations translate molecular insights into treatment strategies that address ER signalling together with major resistance pathways. Ongoing research, including studies from Ishino [51], focused on biomarker-driven patient selection and novel targeted combinations, is essential to further optimize outcomes in hormone-driven cancers. Non-genomic ER and AR signalling also provides a mechanistic rationale for combining endocrine therapy with PI3K/AKT/mTOR pathway inhibitors, because kinase pathway activation can sustain tumour survival even when hormone-receptor signalling is pharmacologically suppressed.

Circulating tumour DNA (ctDNA) monitoring provides an important approach for detecting molecular resistance before radiological progression. In ER-positive/HER2-negative advanced breast cancer, ESR1 mutations commonly emerge during treatment with an aromatase inhibitor plus a CDK4/6 inhibitor and are associated with acquired endocrine resistance. The phase III SERENA-6 trial used serial ctDNA testing every 2–3 months to identify emerging ESR1 mutations in patients without radiological disease progression. Patients with newly detected ESR1 mutations were switched from an aromatase inhibitor to camizestrant while continuing CDK4/6 inhibition. This early-switch strategy significantly improved progression-free survival compared with continuing aromatase inhibitor-based therapy, with median progression-free survival of 16.0 months versus 9.2 months. These findings support ctDNA-guided treatment adaptation as a practical precision-medicine strategy for delaying endocrine resistance progression [17]. 

## Targeting resistance pathways: PI3K/AKT pathway

It is well acknowledged that hormone therapy is used to treat cancer that uses hormones to grow. It is an effective and fairly well-tolerated therapy for hormone-dependent cancers such as hormone receptor-positive breast cancer and prostate cancer. Hormone therapy often provides a better prognosis for patients with hormone-dependent cancers compared to other systemic treatments. However, its effectiveness can be greatly reduced when the body develops resistance to its hormonal agents. This is supported by Browne & Okines [52], who stated that the development of resistance, either intrinsic or acquired, is one of the main challenges in hormonal therapy for ER⁺ breast cancer.

The primary cause of resistance to hormonal therapy is the Phosphoinositide 3-Kinase/Protein Kinase B/Mechanistic Target of Rapamycin pathway or commonly known as PI3K/AKT/mTOR pathway. This pathway’s dysregulation and hyperactivation is often associated with resistance to hormone-based therapies, especially in hormone receptor-positive (HR+) cancers such as breast cancer. According to Rascio et al. [53], hyperactivation of the PI3K/AKT pathway affects carcinogenesis, tumour proliferation, invasion, metastasis, and resistance to some anticancer medications. Taken together, Rascio et al. [53] and Guerrero-Zotano et al. [54] link PI3K/AKT/mTOR hyperactivation to both tumour progression and reduced endocrine sensitivity.

The PI3K/AKT/mTOR pathway plays an important role in our body. It works by regulating many cellular functions in our body, including cellular growth, body metabolism, cell proliferation, protein synthesis, and for body survival, as stated by Jiang et al. [55]. However, if this pathway fails to perform correctly, it results in unintended growth of cells. Consistent with this pathway-level evidence, Hao et al. [56] reported that activating mutations of PIK3CA, which boost P110a activity and encourage excessive cell division and resistance to apoptosis, are present in about 30%–40% of estrogen receptor-positive (ER+) breast tumours. In addition to promoting tumour growth, these changes also may reduce the efficacy of endocrine treatment, which ultimately leads to therapeutic resistance. Therefore, hormone therapy alone may not be beneficial or effective if the main cause of cancer to occur is due to PI3K/AKT/mTOR pathway hyperactivity.

**Fig. 3:**
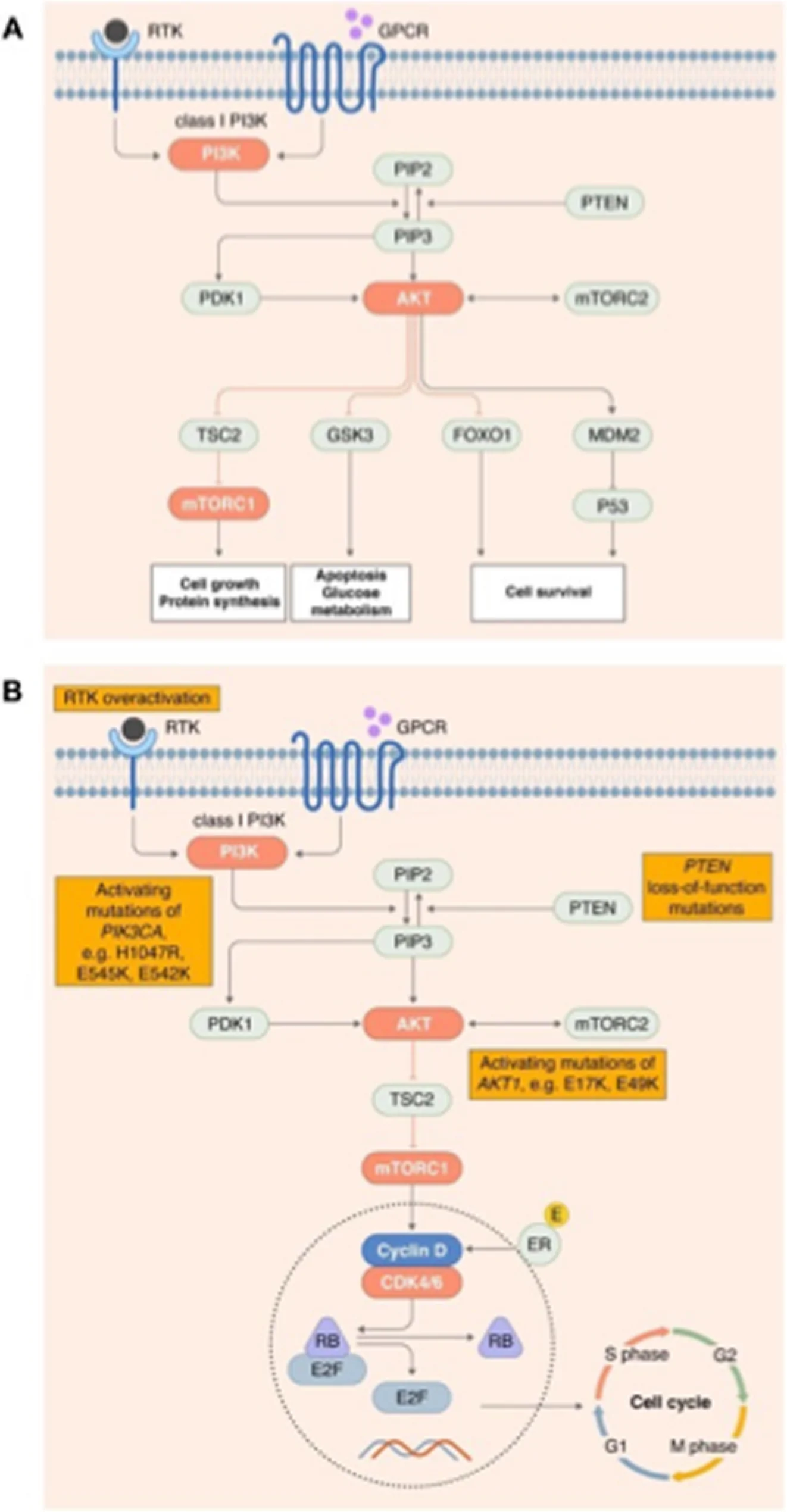
The PI3K/AKT/mTOR pathway (**A**) in normal physiological settings and (**B**) its abnormalities that aid in the development and spread of cancer. Reprinted from [56]

PI3K belongs to the family of membrane-bound lipid kinases. The mechanism of the PI3K pathway was explained in the paper titled *“PI3K/AKT/mTOR inhibitors for hormone receptor-positive advanced breast cancer*,*”* written by Hao et al. [56]. Firstly, the PI3K pathway will be activated by cell surface receptors, for instance, G protein-coupled receptors (GPCRs) and receptor tyrosine kinases (RTKs). When the PI3K receptor is activated, phosphatidylinositol-4,5-biphosphate (PIP2) will be phosphorylated into phosphatidylinositol-3,4,5-triphosphate (PIP3). Next, PIP3 will recruit AKT to the plasma membrane and activate it through phosphorylation by both phosphoinositide-dependent protein kinase 1 (PDK1) and mTOR complex 2 (mTOR2). After that, the fully activated AKT will act on its downstream targets, such as mTOR complex 1 (mTORC1). Hyperactivation of this pathway may contribute to the occurrence of mutations, leading to cancer cell growth (Fig. 3). 

PI3K/AKT/mTOR inhibitors are a class of medication designed to block the hyperactive PI3K/AKT/mTOR signalling pathway. These inhibitors were divided into three different categories depending on each target in the pathway, which are PI3K inhibitors, AKT inhibitors, and mTOR inhibitors. PI3K inhibitors work to prevent the formation of PIP3, which will inhibit AKT activation and downstream survival signals. Examples of PI3K inhibitors in the market are Alpesilib, Inavolisin, Burarlisib, and Piclitisib. Each PI3K inhibitor will be different in its effectiveness against certain types of cancer and their side effects because different agents will inhibit different PI3K enzymes. For instance, in May 2019, the Food and Drug Administration (FDA) approved Alpesilib for the treatment of postmenopausal women and male patients with hormone receptor (HR)-positive, human epidermal growth factor receptor 2 (HER2)-negative, PIK3CA-mutated, advanced or metastatic breast cancer in combination with fulvestrant [57].

In contrast, AKT inhibitors have been developed to block hyperactive AKT signalling. This is supported by evidence showing that amplification and overexpression of AKT2 often relate to increased cancer aggressiveness and poor patient survival, particularly in breast, prostate, ovarian, colorectal, and pancreatic cancers [57]. According to Dong et al. [58] AKT inhibitors are classified into two PH domain inhibitors, which are ATP-competitive inhibitors and allosteric inhibitors, depending on the structure and binding site. However, several PH domain inhibitors cannot be marketed as they are already terminated in the preclinical stage due to low pharmacokinetic properties and high toxicity. Currently, ATP competitive inhibitors such as Capivasertib and Ipatasertib are becoming research-centred. Capivasertib, under brand name TRUQAB, was approved on November 17th, 2023, by the FDA for the treatment of cancer with HR-positive, HER2-negative locally advanced or metastatic breast cancer with one or more alterations in PIK3CA/AKT1/PTEN gene(s) in conjunction with fulvestrant. Ipatasertib is most effective when combined with standard chemotherapy like paclitaxel, carboplatin, and docetaxel or other targeted agents, as it enhances their anti-cancer effects.

Furthermore, mTOR inhibitor agents are also developed in treating cancer, which target the PI3K/Akt/mTOR pathway. Everolimus was indicated for HR+/HER2-breast cancer. It is the first approved mTOR inhibitor due to its demonstrated improvement in PFS. Meanwhile, other mTOR inhibitors such as temsirolimus have not shown sufficient benefit and require further research to determine a suitable patient population [59]. mTOR inhibitors such as everolimus will act by inhibiting mTORC1, suppressing protein synthesis and cellular growth. Since mTOR over-activation is a key mechanism that leads to endocrine resistance, inhibiting mTOR can help restore the sensitivity to hormonal therapy. This explains why the combination of everolimus with exemestane has shown significant clinical benefits. In patients with hormone-receptor-positive advanced breast cancer who had previously received treatment with nonsteroidal aromatase inhibitors, the combination of everolimus and an aromatase inhibitor improved progression-free survival [60].

## Conclusion

Endocrine therapy remains central to the management of hormone-responsive breast and prostate cancers, but receptor alterations, adaptive signalling and pathway crosstalk limit long-term efficacy and support the need for more specific targeted therapeutic approaches.

Oral SERDs offer improved convenience and activity against some ESR1-mutant tumours, but agent-specific differences in efficacy, tolerability and long-term outcomes require individualized patient selection. In contrast to injectable Fulvestrant, oral agents such as Elacestrant, Camizestrant, Giredestrant, and Imlunestrant have demonstrated potential preclinical and clinical performance resulting in improved pharmacokinetic characteristics and increased convenience for patients.

Moreover, oral GnRH antagonists, including Relugolix have solved the significant disadvantages related to traditional GnRH agonists in prostate cancer through giving quick testosterone suppression without the testosterone flare event. Issues with side effects, cost-effectiveness and long-term results continue to become crucial considerations in treatment decision-making, notwithstanding clinical advantages and enhanced cardiovascular safety profiles in certain populations.

Targeted combinations with CDK4/6 and PI3K/AKT/mTOR inhibitors reflect the shift toward biomarker-guided endocrine treatment. The combination of endocrine medications with CDK4/6 inhibitors and PI3K/AKT/mTOR pathway inhibitors proved to enhance progression-free survival (PFS) in certain patient groups, including those with PIK3CA or ESR1 mutations. These methods show a move in approaching precision oncology in which therapy decisions are guided by molecular profiling to overcome resistance and enhance results.

PROTAC-based degraders represent a promising approach for eliminating oncogenic receptors, but their translation into routine care is constrained by delivery, bioavailability, toxicity and resistance. PROTAC-based degraders such as ARD-61 demonstrate high prospects for overcoming resistance in prostate and breast malignancies due to the fact that they eliminate oncogenic proteins rather than simply block their function and activity. In summary, the field of endocrine therapy is currently transforming, including targeted protein degradation technologies and biomarker-driven combination methods replacing single-agent hormone suppression.

## Data Availability

No datasets were generated or analysed during the current study.
